# The burden of *Plasmodium vivax* relapses in an Amerindian village in French Guiana

**DOI:** 10.1186/1475-2875-12-367

**Published:** 2013-10-24

**Authors:** Mathieu Nacher, Aurelia Stefani, Celia Basurko, Delphine Lemonnier, Félix Djossou, Magalie Demar, Narcisse Elenga, Paul Brousse, Muriel Ville, Bernard Carme

**Affiliations:** 1 Centre d’Investigation Clinique Epidémiologie clinique Antilles Guyane (Inserm / DGOS CIE 802), Centre Hospitalier de Cayenne, Cayenne, French Guiana; 2 Epidemiologie Parasitoses et Mycoses Tropicales, EA 3593, Université Antilles Guyane, Cayenne, French Guiana; 3 Pharmacie Centrale, Centre Hospitalier de Cayenne, Cayenne, French Guiana; 4 Unité des Maladies Infectieuses et Tropicales, Centre Hospitalier de Cayenne, Cayenne, French Guiana; 5 Service de Pédiatrie, Centre Hospitalier de Cayenne, Cayenne, French Guiana; 6 Département des Centres délocalisés de prévention et de soins, Centre Hospitalier de Cayenne, Cayenne, French Guiana; 7 Laboratoire Hospitalo-Universitaire de Parasitologie-Mycologie, Centre Hospitalier de Cayenne, Cayenne, French Guiana

## Abstract

Malaria is a public health problem in French Guiana. *Plasmodium vivax* is the most frequent parasite. The objective of this analysis was to estimate the proportion of relapses in the burden of vivax malaria using the statistical rule stating that any case of vivax malaria occurring less than 90 days following a first episode is a relapse.

A total of 622 subjects were followed for 2,9 years with 336 first single episodes of *P. vivax* malaria, and a total of 1,226 episodes of vivax malaria among which 559 were relapses (45.5%). For 194 patients having had falciparum malaria followed by vivax malaria it was estimated that 19% of the vivax episodes occurred less than 90 days following the falciparum episode and thus were possibly relapses due to the activation of latent hypnozoites. Despite the number of vivax cases and the number of relapses, there were only 28 recorded primaquine prescriptions (3.4% of vivax episodes, 4.5% of subjects).

The present study points out that despite the fact that nearly half of the *P. vivax* cases, many of which in children, are caused by latent hypnozoites, only a minority of them benefit from primaquine radical cure. The obstacles to this are discussed and suggestions are made to reduce the burden of vivax malaria in Camopi and other remote health centres in French Guiana.

## Background

Malaria remains a public health problem in French Guiana, a French overseas territory in South America [1-3]. Ninety four percent of the territory is covered by rainforest. Due to a combination of interventions, the visible part of malaria incidence has decreased to a total of around 800 cases a year in 2012. Malaria mostly affects around 30,000 persons living in remote areas and 10,000-15,000 *garimpeiros* living in the forest [4]. Much of the malaria problem originates from this last group that is out of the reach of the health system and makes malaria elimination elusive [5]. Before 2008, there were mines 15 km from Camopi. Since the Harpie operations from the French armed forces started in 2008, the closest mines are at least 30 km from Camopi. Garimpeiros do not stay in Camopi, but they may stay in the neighbouring town across on the Brazilian side. However, some of the persons living in Camopi are involved in supplying mining sites where they may get infected. Between 2001 and 2009, in a cohort of Amerindian children in Camopi, a village on the Eastern border of French Guiana, the overall annual incidence rates of *Plasmodium vivax* malaria were 514 per 1,000 persons for single infections, and 21 per 1,000 persons for mixed infections [1]. The *P. vivax* incidence varied between 300 per 1,000 persons and 935 per 1,000 persons. Recently, data was presented suggesting that, in French Guiana, an interval under 90 days between two *P. vivax* infections was most likely a relapse, whereas over 90 days it was presumed to be a re-infection [6]. In French Guiana, there have been specific logistical and legal difficulties for the effective delivery of a radical cure by targeting hypnozoites with primaquine. The objective of the present study was to better describe the issue of relapses in this cohort, and its potential implications.

## Methods

### Study site

The study took place in Camopi, an Amerindian village in the Amazon forest along the Oyapock River, which delineates the border with Brazil. A retrospective open cohort study was carried out with data from all children born between January 1, 2001 and December 31^st^ 2011. The children come to the health centre approximately once a month, generally for treatment or for prevention (i.e. vaccination). Every six months, a researcher verifies that all newborn children are included in the cohort, that all children are present in the village, and that all the malaria data is collected. Data for children for whom follow-up by the health centre had been interrupted was right censored at the interruption date. Given the isolation of Camopi and the fact that malaria diagnosis and health care is delivered free of charge, it was assumed that all malaria cases were recorded by the health centre.

There has been a recent malaria decline which was concomitant with a number of events: (i) the distribution of rapid diagnostic tests, starting in 2007, clearing the vegetation around homes starting in 2007(2); (ii) the “Harpie operation” involving over 1,000 soldiers and military police, which have, since March 2008, dismantled illegal mining sites, sent back arrested miners to their country, and destroyed on-site mining equipment, with possible effects on malaria transmission; (iii) insecticide-treated bed nets started being distributed in 2010; and (iv) the creation of the regional health agency which led to an intensification of trans-border cooperation.

### Malaria diagnosis

All clinical malaria episodes, their date of occurrence, *Plasmodium* species identification were made in the Camopi health centre. Blood smears were first analysed in Camopi by a trained nurse and then cross-checked by the Parasitology Unit of Cayenne General Hospital, the reference centre for parasitological diagnosis in French Guiana. Malaria was defined as temperature > 38°C at the time of consultation or fever within the past 48 hours associated with *Plasmodium* asexual forms on a thin blood smear (detection threshold: 50 *Plasmodium*/μl). The treatment prescribed was also collected in the medical records.

### Treatment

During the study period, all confirmed cases of *P. vivax* received an unsupervised three-day treatment of chloroquine (total 25 mg/kg). In Camopi, treatment with a combination of primaquine (14 days) and chloroquine (3 days) was supposedly initiated in 2005, following a first relapse. This prescription of primaquine implies respecting French prescription rules: systematic screening for G6PD deficiency (contra-indication if case of G6PD deficiency) this is performed in Cayenne and thus leads to delays in obtaining results; in addition prescription requires a nominative temporary use authorization from the state drug authority, which requires paperwork by the physician and delays between the transmission of the paperwork and the authorization. This procedure has been simplified in 2012 to reduce prescription delays. For primaquine prescription, the data from the medical records was cross-checked by looking at the list of patients having benefitted from a temporary authorization for the use of primaquine.

### Analysis

The number of vivax cases per year was retrieved with a differentiation between relapses according to the 90-day criteria (if malaria occurs between 7 and 90 days following a first episode of vivax, it is a relapse, if it occurs after 90 days it is a new infection). Incidence rates were calculated by age group, and by year using multiple failure models. In order to obtain the relapse rate, a graph was produced based on the fact that the relationship between the numbers of patients who experience one or more, two or more, three or more etc relapses is exponential summarized by the formula x^n^ where x is the fraction of patients experiencing n relapses [7]).

### Ethical and regulatory aspects

All parents were given an explanation and written consent was obtained for the study. The protocol was approved by the CCTIRS (Comité Consultatif pour le Traitement des Informations de Recherche en Santé), and the database was declared to the Commission Nationale Informatique et Libertés, the French regulatory authority.

## Results

There were 622 subjects followed for a total of 2,9 years with 336 first single episodes of *P. vivax* malaria, and a total of 1,226 episodes of vivax malaria among which 559 were relapses (45.5%). For 194 patients having had falciparum malaria followed by vivax malaria it was estimated that 19% of the vivax episodes occurred less than 90 days following the falciparum episode and, thus, were possibly relapses due to the activation of latent hypnozoites.

**Figure 1:**
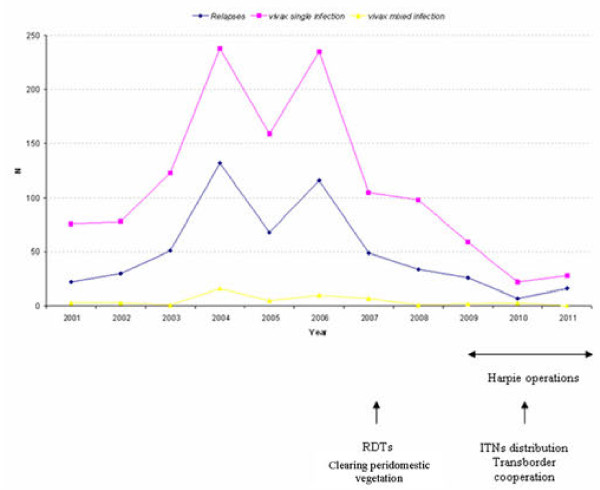
Annual number of vivax malaria cases in Camopi, French Guiana.

**Figure 2:**
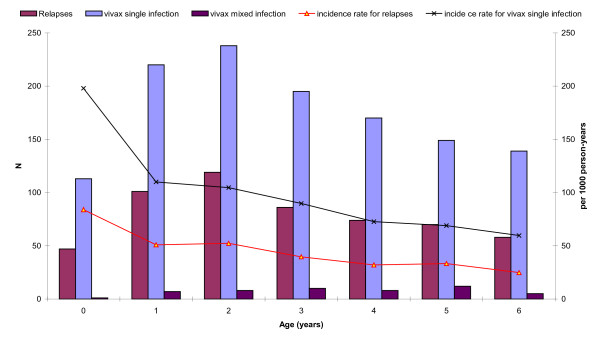
Number of cases and incidence of Plasmodium vivax malaria by age group (2001-2011).

**Figure 3:**
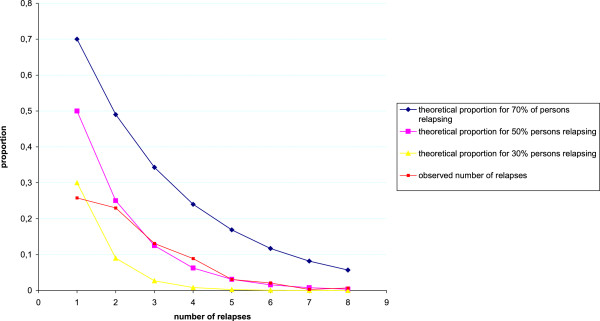
Theoretical proportion of children concerned by N or more relapses.

Figure 1 shows the temporal evolution of vivax malaria and of relapses in Camopi with a recent significant decline of malaria incidence. Figure 2 shows the decline of vivax malaria and relapses according to age. Figure 3 shows the theoretical and observed proportion of children with n or more relapses. This showed that the observed data was closest to the theoretical proportion of 50% of persons relapsing.

For a total of 1,226 *vivax* single infections and 559 relapses according to the 90-day criteria in 622 subjects in Camopi there were 28 recorded primaquine prescriptions (3.4% of vivax episodes, 4.5% of subjects). In 2011-2012, in French Guiana, there were approximately 2,000 malaria cases of which 75% were vivax malaria. During the same period there were 259 prescriptions of primaquine, thus 30% of the number of reported vivax cases.

## Discussion

Although all vivax primary infections do not lead to subsequent relapses, calculations showed that around half would lead to subsequent relapses. Similarly, 19% of patients having had falciparum malaria had a vivax malaria episode within 90 days, suggesting the *P. falciparum* infection could have activated latent hypnozoites [7]. In this remote village, a large proportion of the children’s vivax malaria burden appeared to be due to relapses. A large proportion of the illness and perhaps an even larger proportion of the transmission of vivax malaria are thus attributable to the activation of latent hypnozoites. The striking finding was that despite this, primaquine was very rarely used for a radical cure. Since a consensus conference in 2002, it was recommended in French Guiana, the high council for public health of France also recommended it, notably in endemic areas, at a dose of 0.5 mg/kg/day with 30 mg/day limit for children. However, obviously there are hurdles that stand in the way of the implementation of these recommendations. The G6PD test needs to be performed in Cayenne, samples are shipped by canoe, followed by a three-hour drive to reach the laboratory. This is logistically complicated and leads to delayed results, which is not ideal for patient follow-up. In addition, the extra paperwork required in order to obtain official permission to use this drug and the delays between the demand and the authorization adds more delays, and possibly leads some physicians to avoid the complications. Finally, a number of physicians think that it would be useless to use primaquine in endemic areas and only use it as terminal prophylaxis when patients come back to live in an area without malaria. Overall, these complications probably converge towards the non-use of primaquine, a drug that could theoretically greatly reduce the vivax problem, in Camopi, and other malaria endemic areas of French Guiana.

This sobering observation should stimulate to propose solutions to fix the problem. First, the G6PD problem: Nearly all pregnant women from remote areas deliver in one of the three hospitals in French Guiana, including women from Camopi. Thus, neonates coming from malaria endemic areas could be easily screened for G6PD deficiency before hospital discharge, and their result written in their health book. Another alternative would be point of care testing, with the possibility of using Beutler’s fluorescent spot test on site, or to validate rapid G6PD tests on the field [8,9]. Finally, mass G6PD screening in Camopi, perhaps coupled with active case detection could also allow to have the G6PD results for all persons (Camopi has a population around 1,600). It is perplexing that this old drug (FDA approved in 1952) still does not have official approval in France, 61 years later. The high council for public health could only wish for a pharmaceutical company to take on the burden of applying for approval by the authorities. Given the rarity of malaria in France, there is no market, and no incentive for pharmaceutical companies to start the approval process.

A large portion of the vivax malaria problem in these Amerindian children is linked to the activation of latent hypnozoites. Despite research aiming to differentiate primary infections from relapses [6,10], despite recommendations to use primaquine, this reality does not lead to changes on the field. Malaria eradication is challenging in this remote area surrounded by the Amazon forest and often in contact with illegal gold miners who represent uncontrolled transmission reservoirs [11]. This is compounded by vivax relapses and the absence of radical cure. There are some solutions to facilitate the implementation of standard treatment policy. Authorities should do all they can to facilitate the prescription of primaquine in French Guiana. With optimal adherence ensured by supervised administration in this small village, radical treatment will improve the situation of a number of individual patients. New drugs, such as tafenoquine, may improve compliance and new tests such as field adapted RDT for G6PD may facilitate prescription.

## Competing interests

The authors declare that they have no competing interests.

## Authors’ contributions

MN wrote the first draft & analyzed the data. AS BC DL provided data. MN AS CB MD DL PB MV BC participated in the data interpretation and final manuscript. All authors read and approved the final manuscript.

## References

[B1] Stefani A Hanf M Nacher M Girod R Carme B Environmental, entomological, socioeconomic and behavioural risk factors for malaria attacks in Amerindian children of Camopi, French Guiana Malar J 2011 12 246 10.1186/1475-2875-10-246 21861885 PMC3196925

[B2] Hustache S Nacher M Djossou F Carme B Malaria risk factors in Amerindian children in French Guiana Am J Trop Med Hyg 2007 12 619 625 17426159

[B3] Carme B Substantial increase of malaria in inland areas of eastern French Guiana Trop Med Int Health 2005 12 154 159 10.1111/j.1365-3156.2004.01365.x 15679558

[B4] Tabor D Like butterflies in the jungle: The quest for the new El Dorado Harpers Magazine 2011 12 45 54

[B5] Nacher M Guerin PJ Demar-Pierre M Djossou F Nosten F Carme B Made in Europe: will artemisinin resistance emerge in French Guiana? Malar J 2012 12 152 23641802 10.1186/1475-2875-12-152 PMC3649934

[B6] Hanf M Stephani A Basurko C Nacher M Carme B Determination of the Plasmodium vivax relapse pattern in Camopi, French Guiana Malar J 2009 12 278 10.1186/1475-2875-8-278 19961585 PMC2793261

[B7] White NJ Determinants of relapse periodicity in *Plasmodium vivax* malaria Malar J 2011 12 297 10.1186/1475-2875-10-297 21989376 PMC3228849

[B8] Tantular IS Iwai K Lin K Basuki S Horie T Htay HH Matsuoka H Marwoto H Wongsrichanalai C Dachlan YP Kojima S Ishii A Kawamoto F Field trials of a rapid test for G6PD deficiency in combination with a rapid diagnosis of malaria Trop Med Int Health 1999 12 245 250 10.1046/j.1365-3156.1999.00395.x 10357861

[B9] Meissner PE Coulibaly B Mandi G Mansmann U Witte S Schiek W Müller O Schirmer RH Mockenhaupt FP Bienzle U Diagnosis of red cell G6PD deficiency in rural Burkina Faso: comparison of a rapid fluorescent enzyme test on filter paper with polymerase chain reaction based genotyping Br J Haematol 2005 12 395 399 10.1111/j.1365-2141.2005.05778.x 16225660

[B10] Veron V Legrand E Yrinesi J Volney B Simon S Carme B Genetic diversity of msp3alpha and msp1_b5 markers of *Plasmodium vivax* in French Guiana Malar J 2009 12 40 10.1186/1475-2875-8-40 19284592 PMC2660359

[B11] Van den Eede P Soto-Calle VE Delgado C Gamboa D Grande T Rodriguez H Llanos-Cuentas A Anné J D'Alessandro U Erhart A *Plasmodium vivax* sub-patent infections after radical treatment are common in Peruvian patients: results of a 1-year prospective cohort study PLoS One 2011 12 e16257 10.1371/journal.pone.0016257 21297986 PMC3030575

